# A Knowledge, Attitude, and Practice (KAP) Study on Phlebotomy Among Nurses in a Tertiary Hospital in Patna, India

**DOI:** 10.7759/cureus.50372

**Published:** 2023-12-12

**Authors:** Soma Dey, Neha Rai, Akash Bansal, Bandana Kumari, Bankim Das, Alka Kumari

**Affiliations:** 1 Biochemistry, All India Institute of Medical Sciences (AIIMS) Patna, Patna, IND; 2 Biochemistry, All India Institute of Medical Sciences (AIIMS) Gorakhpur, Gorakhpur, IND; 3 Transfusion Medicine, All India Institute of Medical Sciences (AIIMS) Patna, Patna, IND

**Keywords:** attitude, knowledge, phlebotomy, nurses, needlestick injuries

## Abstract

Introduction: For most clinicians and nursing officers, laboratory testing is an unfamiliar part of medical caregiving, and ignorance may lead to serious avoidable errors. Phlebotomy, the first basic step towards laboratory testing, is to be taken seriously otherwise unnecessary repeat testing becomes mandatory. We hypothesized that there are some gaps in knowledge, attitude, and practice (KAP) among these nursing officers regarding practices of phlebotomy, which may influence the quality of blood samples. This study aims to assess the overall nurses’ knowledge, attitude, and practice of phlebotomy to provide a remarkable improvement in blood sampling practice in our hospital.

Material and methods: A prospective study was conducted involving nurses posted in different wards in All India Institute of Medical Sciences (AIIMS) Patna, India. A phlebotomy questionnaire was designed based on KAP on the clinical and laboratory standards of the WHO guidelines. A total of 30 questions were distributed among the nursing staff, 10 each from knowledge, attitude, and practice. Descriptive and logistic regression analyses were used to analyze the KAP levels and their influencing factors. All continuous variables were tested for normality conditions using the Shapiro-Wilk test and P>0.05 were considered for normality.

Results and conclusion: The total average score of knowledge among the nurses was 7.62 (95%CI: 15.77-16.56). It was found that the nurses, on average, had a very strong positive attitude (93.36%). Regarding the distribution of practices of nurses, it was found that 87% had good practice as most of the positive practice items had high responses. The knowledge of phlebotomy among nurses was found to be satisfactory, except in a few areas. An education program on phlebotomy should be developed for nurses to improve and enhance their knowledge of phlebotomy.

## Introduction

Laboratory services have become an integral part of the modern healthcare sector, and venous blood collection is one of the most common invasive procedures. Even though it is thought to be a safe procedure, it is associated with its risks such as needle stick injuries and preanalytical errors [1,2]. To perform successful phlebotomy, both theoretical knowledge, as well as practical skills, are required [3]. Lack of knowledge or skills may lead to many errors in the procedure which might result in altered results and add to patient suffering whereas wrong lab results can affect the further treatment and diagnosis of the patient and affect patient care [4]. It can lead to the exposure of patients and healthcare workers to various infectious blood-borne diseases [5].

Laboratory processing of samples is divided mainly into three steps: preanalytical, analytical, and postanalytical. Preanalytical steps include patient preparation, collection, and handling of blood specimens. Errors that affect laboratory test results mostly occur in the preanalytical phase, primarily due to the lack of standardized procedures for sample collection [6]. Since a large proportion (60-80%) of medical decisions are evidence-based and directly depend on the results of the laboratory tests, any error in the above-mentioned steps can cause serious consequences, primarily for the patients but also for medical professionals and the healthcare system in general, as repeated testing can lead to wastage of infrastructure, time, and money [7]. In addition, there are large differences in the educational qualifications of medical staff performing phlebotomy (nurses, laboratory technicians, phlebotomists), which adds another layer of variation to the system [8]. Therefore, to minimize the chances of errors, there should be an evaluation of the phlebotomy knowledge among the staff who are performing the procedure.

Some European and African studies reveal a lack of adequate knowledge among healthcare professionals about the correct phlebotomy practices. The rate of correct patient identification, wearing gloves, tourniquet application time, order of draw, and mixing of tubes have been found to be unacceptably low [9]. The World Health Organization (WHO) has developed various such guidelines in an attempt to reduce the rate of errors in the phlebotomy process and also protect the health of workers from any injury or infectious diseases [10]. There are very few studies available that assess the quality of venous blood sampling and the knowledge of phlebotomists carrying out the procedure [2].

At our institute, phlebotomy for patients who are admitted to different wards is conducted by nursing officers. There is very little systematic training offered on blood specimen quality control and sample collection practices in Indian nursing education [11].

## Materials and methods

A prospective study was conducted at the All India Institute of Medical Sciences (AIIMS) in Patna, Bihar, India. A total of 119 participants (nurses) who were posted in different wards were included in the study. A questionnaire with 30 questions was designed based on the knowledge, attitude, and practices (KAP) related to venous blood sampling and the clinical laboratory based on standard WHO guidelines [12] and consultation with researchers who had conducted KAP surveys elsewhere. The questionnaire was delivered by Google Forms (Google LLC, Mountain View, California, United States). The nurses completed the questionnaires anonymously and submitted them to the investigators within one week. No incentives were offered for participation. The Institute Ethical Committee, AIIMS Patna approved the study (approval number: AIIMS/Pat/IEC/2021/669). Informed consent was obtained from each investigated nurse.

The final version of the questionnaire comprised three sections: (i) demographic profiles of the nurses such as age, education, hospital, department, and professional title, (ii) KAP questions (including single-choice questions, multiple-choice questions, and fill-in-the-blanks) on venous blood sample collection, and (iii) issues from pre-sampling, sampling, and post-sampling phases. The questions were validated by experts who independently rated them as satisfactory or unsatisfactory. The experts offered some suggestions which were incorporated and sent for further approval.

Statistical analysis

After proper coding of the variables, all data were entered into MS Excel (Microsoft Corporation, Redmond, Washington, United States). All data were analyzed using Stata Statistical Software: Release 10 (2007; StataCorp LL., College Street, Texas, United States). All continuous variables were tested for normality conditions using the Shapiro-Wilk test and P>0.05 were considered for normality. Continuous variables were presented as mean with standard deviation for normal data, otherwise presented as median with interquartile range. Categorical variables were presented as proportions. A two-sample t-test with equal variances was applied to compare the mean score of KAP between male and female nurses. Mann-Whitney Wilcon test was used for comparing equality of distribution for skewed variables. Linear regression was performed to assess the effect of some independent variables like sex, age, and educational status on the level of KAP. Cronbach’s alpha was estimated for variables like KAP to assess the consistency of items within each variable.

## Results

A total of 119 participants responded to the questionnaire. Table 1 presents the sociodemographic characteristics of the participants. Out of the total 119 respondents, 73 (61%) were females. Approximately 78% had completed a B.Sc. or B.Sc (honors) Nursing, and 111 (93%) were working as Nursing Officers.

**Table 1:** Sociodemographic distribution of the nurses (N = 119) GNM: General Nursing and Midwifery; HDU: High-Dependency Unit; HICC: Hospital Infection Control Committee; BB: B2 ward in the Study Hospital; GN: Graduate Nurse; RN: Registered Nurse

Variables	Frequency	Percentage
Gender		
Female	73	61.34%
Male	46	38.66%
Age Group		
< 26 years	38	31.93%
26-35 years	79	66.39%
>35 years	2	1.68%
Educational Qualification		
GNM/Intermediate	19	15.97%
B.Sc. Nursing	93	78.15%
M.Sc. Nursing	7	5.88%
Designation		
Senior Nursing Officer	2	1.68%
Nursing Officer	111	93.28%
Staff Nurses (GN/RN)	6	5.04%
Department		
Medical Ward	19	15.9%
Surgical Ward	19	15.9%
Obstetrics and Gynecology	1	0.84%
Trauma and Emergency	13	10.9%
ICUs	19	15.9
Others (HDU, HICC, BB, Triage)	48	40.3

Table 2 presents the distribution of knowledge of nurses, and it was found that the nurses, overall, had a good knowledge of phlebotomy as 78.3% gave correct responses to the items related to knowledge. The total knowledge score was tested for the normality using Shapiro-Wilk test and it was found to be normally distributed (p= 0.8268). The total average score of knowledge among the nurses was 7.62 (95%CI: 15.77-16.56). The average score of knowledge of phlebotomy among the female nurses was 7.72 (95%CI: 7.43-8.02) in comparison to male nurses with a mean score of 7.45 (95% CI: 7.09-7.81), with no statistically significant difference (t-test statistic = 1.14, p=0.253). Linear regression analysis showed that knowledge level decreased per unit increase in age (beta coefficient= -0.027, p=0.423) but was not significant, and knowledge score increased as the educational qualification increased but was not significant (beta coefficient = 0.135, p=0.164). Internal consistency of knowledge items was tested using Cronbach alpha coefficient, and it was found to be 0.3373 with two negative items showing very average consistency in items. The percentage score was categorized into three categories: ≥75% correct response, 50-74% correct response, and <50% correct response. Nearly 62% of female nurses had more than 75% correct responses as compared to 48% of male nurses.

**Table 2:** Distribution of knowledge about phlebotomy among the nurses * p value < 0.05 is significant CR No.: Central Registration Number

Item No.	Item	True, n (%)	False, n (%)	Mean/p-value
1	Patient identity be confirmed prior to blood sampling with at least two checks, i.e., name and CR no.	119 (100%)	0 (0%)	7.62/p=0.253
2	The proper angle for needle insertion is >45⁰	68 (57.14%)	51 (42.86%)	
3	If the required sample tube is not available in the ward, then the sample can be sent in a tube with a different color	112 (94.12%)	7 (5.88%)	
4	Samples for glucose estimation can be sent in a tube with the red-color cap	106 (89.08%)	13 (10.92%)	
5	If sampling is required in multiple vacutainers, sampling should be done with a needle holder and adapter	89 (74.79%)	30 (25.21%)	
6	The puncture site can be palpated with a finger by the phlebotomist after disinfecting the area	89 (74.79%)	30 (25.21%)	
7	Needle and syringe should be discarded immediately into a robust sharps container	101 (84.87%)	18 (15.13%)	
8	Sample for detection of hemoglobin variants can be sent after a blood transfusion	47 (39.5%)	72 (60.5%)	
9	It is not necessary to check the gauge size of the needle before withdrawing blood	68 (57.14%)	51 (42.86%)	
10	A blood sample can be drawn from the hand receiving IV fluids	108 (90.76%)	11 (9.24%)	

Table 3 presents the distribution of attitudes of nurses, and it was found that the nurses, overall, had a very strong positive attitude, with 93.36% giving responses in the categories of “Strongly agree” or “Agree”, which were correct, as compared to “Strongly disagree” or “Disagree”. Shapiro-Wilk test was applied to test the normality of the total attitude score of the nurses, and it was found to be not normally distributed (p=0.0002). The total attitude score ranged from 0 to 30. The median attitude score in female nurses was 27 (interquartile range (IQR): 25-28); however, there was no significant difference (Mann-Whitney Wilcoxon Test, z= -0.490, p=0.6245). Linear regression analysis was performed to assess the effect of age on attitude, and it was found that for per unit increase in age, the attitude score decreased by an amount of 0.152 (beta coefficient = -0.152, p = 0.067), but not significant. Linear regression analysis was also performed to assess the effect of the educational status of nurses on attitude, and it was found that for a per unit increase in the level of educational status, the attitude score increased by an amount of 0.217 (beta coefficient = 0.217, p = 0.366), but this was not significant. The reliability coefficient was found to be very consistent among the items of attitude measurement (Cronbach’s alpha = 0.70) among the nurses. The percentage score was categorized into three categories: ≥75% positive response, 50-74% positive response, and <50% positive response. Nearly 87% of male nurses had more than 75% positive responses as compared to 85% of female nurses, but the difference in attitude between female and male nurses was not much.

**Table 3:** Distribution of attitude about phlebotomy among the nurses p-value < 0.05 is significant ABG: Arterial Blood Gas; CR No.: Central Registration Number

S. No.	Item	Response				Median ± IQR/p-value
		Strongly Agree (3), n (%)	Agree (2), n (%)	Disagree (1), n (%)	Strongly Disagree (0), n (%)	27 ± 25-28/p = 0.62
1	The sample should be correctly matched with the patient with name, CR No., and sample number before being sent to the laboratory	110 (92.4%)	8 (6.72%)	0 (0%)	1 (0.84%)	
2	Is it necessary to wear well-fitting non-sterile gloves before performing phlebotomy?	45 (37.82%)	38 (31.93%)	23 (19.33%)	13 (10.92%)	
3	Assessment of the patient such as the use of medications, diet restrictions, strenuous exercise, or history of needle syncope should be included before blood sampling	47 (39.5%)	52 (43.7%)	19 (15.97%)	1 (0.84%)	
4	Median cubital vein is the most preferred vein for venepuncture	74 (62.18%)	43 (36.13%)	1 (0.84%)	1 (0.84%)	
5	Pre-heparinized syringes should be used for ABG sampling	109 (91.60%)	8 (6.72%)	1 (0.84%)	1 (0.84%)	
6	Sampling in correctly color-coded sample tubes is important for proper reports of patients	105 (88.24%)	14(11.76%)	0 (0%)	0 (0%)	
7	Formal training and practice for phlebotomists can be useful	82 (68.91%)	34 (28.57%)	2 (2.52%)	1 (0.84%)	
8	Quick transportation of ABG samples in tightly-capped syringes will improve the quality of results from laboratory testing	93 (78.15%)	24 (20.17%)	2 (1.68%)	0 (0%)	
9	It is mandatory to fill vacutainers up to the mark indicated on the tubes	96 (80.67%)	22 (18.49%)	1 (0.84%)	0 (0%)	
10	Blood for the coagulation study should be collected in a light blue-capped tube	95 (79.83%)	17 (14.29%)	1 (0.84%)	6 (5.04%)	

Table 4 presents the distribution of practices of nurses, and it was found that 87% of the nurses had good practice considering the number of correct answers. Shapiro-Wilk test was applied to test the normality of the total score of practice, and it was found to be normally distributed (p=0.236). The average total score of practice was 16.16 (95%CI: 15.77-16.56). There was no significant difference in the mean total score of practice between female nurses having a score of 16.41 (95%CI: 15.93-16.88) in comparison to male nurses with 15.78 (95%CI: 15.07-16.48) (p=0.1225); this was not significantly different (t-test statistic = 1.54, p=0.1225). Linear aggression analysis showed a significant association of qualification with the practice of nurses (beta coefficient = 0.504, t-stat= 3.09, p=0.002), but not with the age of the nurses. The percentage score was categorized into three categories: ≥75% correct response, 50-74% correct response, and <50% correct response. Nearly 84% of female nurses had more than 75% correct responses as compared to 74% of male nurses, indicating a 10% difference in attitude between female and male nurses. The reliability coefficient was found to be consistent among the items of practice (Cronbach’s alpha = 0.5275) among the nurses.

**Table 4:** Distribution of practices in phlebotomy among the participants p-value < 0.05 is significant

S.No.	Items	Response			
		Always (2), n (%)	Sometimes (1), n (%)	Never (0), n (%)	Mean 16.16/p=0.1225
1	Do you check the sample tubes as per color coding before filling the tubes?	119 (100%)	0 (0%)	0 (0%)	
2	Do you sanitize your hands with soap or alcohol rub before withdrawing every sample?	116 (97.48%)	3 (2.52%)	0 (0%)	
3	Do you release the tourniquet as soon as blood flow is established?	98 (82.35%)	11 (9.24%)	10 (8.40%)	
4	Do you dispose of the needle in a puncture-proof sharps container?	118 (99.16%)	0 (0%)	1 (0.84%)	
5	Do you label the blood collection tube after filling the tube?	35 (29.41%)	13 (10.92%)	71 (59.7%)	
6	Do you push blood through the needle into vacutainers?	36 (30.25%)	34 (28.57%)	49 (41.2%)	
7	Do you collect the blood samples from existing peripheral venous access in a hospitalized patient?	58 (48.74%)	26 (21.85%)	35 (29.41%)	
8	Do you allow the area to dry before pricking?	115 (96.64%)	3 (2.52%)	1 (0.84%)	
9	Do you properly mix the blood containing additives by inverting the tube up and down?	111 (93.28%)	7 (5.88%)	1 (0.84%)	
10	Do you decant/pour one tube into another?	105 (88.24%)	5 (4.20%)	9 (7.56%)	

## Discussion

In the present study, 61% (n=73) of respondents were females and nearly 93% (N = 111) were qualified and trained nursing professionals working as Nursing Officers. As per this study, the knowledge of nurses regarding phlebotomy was found to be good as 78.3% (n=94) of the nurses gave correct responses to the questions related to knowledge. In a study by Cai et al. conducted in China, the level of knowledge on phlebotomy among nurses was not found to be quite satisfactory [9]. In another study conducted by Adiga et al. in 2017, the majority of the questions assessing the knowledge domain were correctly answered and no significant difference was found in the knowledge level, between the groups based either on age or number of years of experience [2]. Correct patient identification is crucial to ensure patient safety and proper patient identification must rely on at least two independent identifiers. In this study, nearly 99.2% (n=118) of respondents had correct knowledge about patient identification before blood sampling to reduce preanalytical errors. However, only 57.1% (n= 68) could give the correct answer for the angle of needle insertion. The WHO guideline states that entry of the needle in the vein should be done swiftly at a 30-degree angle or less [12]. Also, only 39.5% (n=47) could correctly answer the question stating that samples for hemoglobin variants can be sent after blood transfusion.

In the present study, a very strong positive attitude was seen as 93.36% (n= 112) of the nurses had given responses in the category of “Strongly agree” or “Agree” as compared to “Strongly disagree” or “Disagree”. The median attitude score in female and male nurses was 27, which was statistically insignificant. It was found in the study that 87% (n= 104) of the nurses had good practice as most of the positive practice items had high responses in the category of “Always”. There was no significant difference in the mean total score of practice between female nurses as compared to male nurses. Nearly 84% (n=64)of female nurses had more than 75% correct responses. The WHO guidelines suggest that the tourniquet should be removed as soon as blood flow is established. In the current study, 82.3% (n= 98) responded that they always release the tourniquet as soon as blood flow is established. There was no significant difference in the mean total score of practice between female nurses to that of male nurses.

Based on the current study, it is seen that the nurses working in our hospital are well-qualified and have a good knowledge of phlebotomy as most of the questions related to knowledge of phlebotomy were answered correctly.

There are some limitations in this study. First, the questionnaire for investigation was self-designed without undergoing a thorough reliability and validity test. However, the references had been taken from WHO phlebotomy guidelines. It is recommended that a standardized questionnaire be developed to investigate the blood sampling knowledge among nurses in the future. Second, the study assesses nurses' overall KAP on phlebotomy in a single center with a small sample size, and in the future, it would be worthwhile to explore factors that influence the KAP level in a large sample size. Third, it is considered that there is a gap in KAP behavior, so it is highly recommended that an observational study for the blood sampling practice among nurses and phlebotomists be conducted to further assess their compliance with guidelines. Continuous medical education and workshops in small batches of 30-50 nursing officers, interns, post-graduate students, and resident doctors are recommended as part of the learning-teaching curriculum at periodic intervals. Thus, we believe that this survey provided important baseline data for understanding the current scenario of phlebotomy KAP among the nurses in tertiary institutes like ours.

## Conclusions

The KAP of phlebotomy among nurses at our hospital is good but still, there is much room to improve. It is very important to raise awareness about the various causes of hemolysis during sample collection and the factors that contribute to preanalytical errors during sample collection. Therefore, intervention in the form of training programs is required. Cooperation and communication between the nursing department and the laboratory should be encouraged to develop increased accuracy in phlebotomy among nurses so that the quality control of blood samples could be improved.
